# Pattern of systemic antibiotic use and potential drug interactions: evaluations through a point prevalence study in Ankara University Hospitals

**DOI:** 10.3906/sag-2004-164

**Published:** 2021-04-30

**Authors:** İrem AKDEMİR KALKAN, Güle ÇINAR, Aysel PEHLİVANLI, Fatih ÜRKMEZ, İzel Ezgi TOPALOĞLU, Büşra AKYOL, Arzu ONAY BEŞİKÇİ, Alpay AZAP, Kemal Osman MEMİKOĞLU

**Affiliations:** 1 Department of Infectious Diseases and Clinical Microbiology, Faculty of Medicine, Ankara University, Ankara Turkey; 2 Department of Pharmacology, Faculty of Pharmacy, Ankara University, Ankara Turkey

**Keywords:** Antibacterial agents, drug interactions, pharmacist

## Abstract

**Background/aim:**

Most of the hospitalized patients are on a number of drugs for comorbidities and/or to prevent nosocomial infections. This necessitates a careful consideration of drug interactions not only to avoid possible toxicities but also to reach the highest efficiency with drug treatment. We aimed to investigate drug interactions related to systemic antibiotic use and compare three different databases to check for drug interactions while characterizing the main differences between medical and surgical departments.

**Materials and methods:**

This point prevalence study covered data on 927 orders for patients hospitalized between June 3 and 10, 2018 in Ankara University Hospitals. Systemic antibiotic use and related drug interactions were documented using UptoDate, Drugs, and Medscape and comparisons between the departments of medical and surgical sciences were made.

**Results:**

The number of orders, or the number of drugs or antibiotics per order were not different between the medical and surgical sciences departments. A total of 1335 antibiotic-related drug interactions of all levels were reported by one, two, or all three databases. UptoDate reported all common and major interactions. Pantoprazole was the most commonly prescribed drug and appeared in 63% of all orders. Among 75 different molecules, ceftriaxone and meropenem were the two most prescribed antibiotics by the surgical and medical departments, respectively.

**Conclusion:**

A dramatic variance existed amongst antibiotics prescribed by different departments. This indicated the requirement for a centralized role of an infectious diseases specialist. Especially for the hospitalized patient, prophylactic coverage with at least one antibiotic brought about a number of drug interactions. A precise evaluation of orders in terms of drug interactions by a clinical pharmacist (currently none on duty) will reduce possible drug-related hazards.

## 1. Introduction

In addition to lack of new antibiotics, the well-recognized danger of resistance to available drugs necessitates rational use of antibiotics in both hospital and outpatient settings. A retrospective study by Centers for Disease Control and Prevention (CDC) reported that between 2006 and 2012, 55% of patients used at least one dose of an antibiotic during their hospital stay [1]. 

Moreover, community-acquired and nosocomial infections require multidrug treatment in most hospitalized patients. The clinicians cannot take the risk not reaching the maximum efficiency of the antibiotic drug. This becomes even more pronounced when so-called “last-resort” antibiotics are used. Considering that most of the hospitalized patients are on a number of drugs for comorbidities, possible drug interactions become one of the crucial aspects of therapy that the clinician is forced to take into account when planning the treatment. One problem for this kind of assessment in developing countries is the affordability of drug databases that are integrated into an interaction tool. In addition, there is little agreement among commonly used drug interaction databases and a gold-standard reference is absent [2]. 

For example, when mixed together in solution in vitro, extended spectrum penicillins result in an inactivation of aminoglycosides [3–6]. A similar inactivation seems to be present in vivo in patients with renal dysfunction and results in a decrease in the half-life of aminoglycoside [7–9]. Hence, the web-based interaction tool UptoDate prompts the clinician to consider therapy modification (Risk Level D) based on references 3–9 when the possible interaction between piperacillin and systemic gentamicin were analyzed [10]. Prescribing information of a piperacillin-containing drug also reports both in vivo and in vitro interactions [11]. On the other hand, another commonly used tool for interactions, Medscape reports that piperacillin increases the effects of gentamicin by pharmacodynamic synergism [12]. 

Interaction between drugs may also result in severe clinical cases such as coagulation abnormalities, organ dysfunctions, or electrolyte imbalances simply due to additive adverse effects. Concurrent use of cotrimoxazole and any inhibitor of the renin-angiotensin-aldosterone system is expected to increase the risk of hyperkalemia, an interaction unequivocally documented in many case reports, as well as a population-based study that linked this combination to sudden death in older patients due to unrecognized hyperkalemia [13]. Thus, a thorough review of the available evidence is required when planning drug treatment to achieve the maximum efficacy while avoiding interaction-related treatment failure or adverse effects.

Studies show that both the quantity and selection of antibiotics still vary dramatically even among hospitals within a country [14] and this is true for Turkey. In addition, Turkey is one of the countries with the highest antibiotic consumption per capita and suffers widespread antimicrobial resistance [15,16].

We aimed to investigate potential drug interactions related to systemic antibiotic use and compare three different databases in documenting these interactions. We also compared the patterns of antibiotic use between the medical and surgical departments of Ankara University Hospitals through this prevalence study.

## 2. Materials and methods 

This study was conducted at Ankara University Hospitals that have a total number of 2000 beds over two campuses and approach an occupancy level of nearly 95% at all times. It was approved by the Ethics Committee of the Faculty of Medicine of Ankara University (28 May 2018, 0958818) with a waiver of informed consent due to the retrospective nature of the study. The data were collected from patients ≥18 years that were hospitalized between June 3 and June 10, 2018. All patients who received at least one systemic antimicrobial (antibacterial, antifungal, or antiviral) drug were included. Demographic data, preexisting medical conditions, and drug regimens were collected via the patient medical record system of Ankara University Hospitals, Avicenna. Antibiotic use was categorized as empiric, prophylactic, and targeted. Empiric use was against an anticipated cause of the infection whereas targeted or definitive use was directed against a known pathogen. Prophylactic use is defined as cases where a clear indication was missing. Potential drug interactions were analyzed using three different databases, UptoDate, Drugs, and Medscape. All three databases have web-based tools to evaluate drug interactions based on the information collected by their expert panels. UptoDate and Drugs, but not Medscape, provide the references for the reported interactions as well. They are also slightly different in terms of the levels of reported interactions. UptoDate reports drug interactions on five levels: X (avoid combination), D (consider therapy modification), C (monitor therapy), B (no action needed), and A (no known interaction). Drugs reports drug interaction on three main levels: major (subdivided into “contraindicated” and “monitor closely”); moderate, and minor. Medscape reports four levels of drug interactions: contraindicated, serious-use alternative, monitor closely, and minor.

Some characteristics related to drug usage were compared between departments of medical and surgical sciences. Next, the efficiency of databases was compared for reporting common, as well as major interactions. To do this, all interactions of all levels that include at least one antibiotic were documented and unique interactions were identified. Each database was evaluated in two ways: 1. documenting the interaction that is reported by all three databases, 2. documenting a major interaction that is reported by all three databases.

The difference between the medical and surgical departments was analyzed using Student’s t-test when data expressed as mean ± SEM were compared. Chi-square test of probability was utilized when comparing medical and surgical departments for empiric, prophylactic, and targeted use of antibiotics. A level of probability of P < 0.05 was deemed to constitute the threshold for statistical significance.

## 3. Results 

A total of 927 orders were collected during the study period. However, only the orders that included at least one systemic antibiotic were evaluated. A total of 907 patients had at least one antibiotic in their orders. Of these patients, 498 were in medical and 409 in surgical clinics. Demographic data and comorbidities are shown in Table 1. Briefly, the median age was 58 years (range 18–97 years) and 481 (53%) patients were female. The most common underlying medical condition was hypertension (312 (34%)).

**Table 1:** Demographic data and comorbidities.

Parameter	Value
Number of patientsMedical ClinicsSurgical Clinics	498409
Age, median (range)	58 (18–97)
Female/male	481/426
Comorbidities, number (%) Hypertension Diabetes Malignancy Renal insufficiency Liver failure Other (Heart failure, coronary artery disease, benign prostate hypertrophy, psychiatric disease, neurologic disease, chronic obstructive pulmonary disease, transplantation)	312 (34) 222 (24) 232 (26) 73 (8) 39 (4) 544 (60)

The main characteristics of drug use are shown in Table 2. The total number of orders, all drugs, and antibiotics were similar between surgical and medical clinics. The percentage of antibiotics to all drugs was not different between the clinics of medical and surgical departments. Somewhat surprisingly, the mean number of orders that included at least one antibiotic was nearly the same between medical and surgical clinics.

**Table 2:** Main characteristics of drug use.

Parameter	Medical clinics,Mean ± SEM	Surgical clinics,Mean ± SEM	P
Number of orders	25 ± 4	26 ± 5	0.92
Number of orders that include an antibiotic	13 ± 2	18 ± 4	0.31
Number of all drugs	249 ± 39	180 ± 36	0.22
(Antibiotic/order) %	11.80	15.13	0.09

A more detailed analysis of drug use is shown in Table 3. The most commonly prescribed drug in all clinics was a proton pump inhibitor (PPI), pantoprazole, which appeared in more than 60% of all orders. The most prescribed antibiotic in the orders from medical clinics was meropenem and appeared in 12% of these orders. This drug was prescribed to only 5% of surgical clinics’ patients. Ceftriaxone was the most common antibiotic in the orders from surgical clinics. Ceftriaxone was prescribed to 17% of patients in surgical clinics and to only 5% of patients in medical clinics.

**Table 3:** Drug use, detailed analysis.

	Medical clinics	Surgical clinics
Most commonly used drug, count	Pantoprazole, 325	Pantoprazole, 255
Two most frequently prescribed antibiotics, count	Meropenem, 62	Ceftriaxone, 72
Number of antibiotics (%) Prophylaxis Empiric Targeted	65 (15) 121 (28) 245 (57)	159 (40) 126 (31) 114 (29)

Types of antibiotic treatment are summarized in Table 4. Medical and surgical clinics were similar in empiric usage of antibiotics. Expectedly, when compared to medical clinics, surgical clinics primarily prescribe antibiotics, namely ceftriaxone and metronidazole, for prophylaxis. In fact, prophylactic use of metronidanazole appeared in 13% of orders from surgical clinics. On the other hand, targeted utilization of antibiotics was higher in medical clinics compared to surgical clinics as opposed to our predictions.

**Table 4:** Type of antibiotic treatment.

Type of therapy	Medical clinics (%)	Surgical clinics (%)	P
Prophylaxis	4.47	33.45	0.005
Empiric	37.80	30.66	0.08
Targeted	57.72	45.94	0.005

A total of 1335 antibiotic-related drug interactions of all levels were reported by one, two, or all three databases. Interactions reported by each database were compared between medical and surgical clinics. First, interactions of all levels per order were compared using each database for medical and surgical clinics. Next, these clinics were compared in terms of interactions of a similar rank of a particular database per order. For example, UptoDate reports the highest level of interactions with “X (avoid combination)” and the second level of interactions with “D (consider therapy modification)” whereas the highest two levels for Drugs are “major-contraindicated” and “monitor closely”. Neither all interactions nor interactions of equal level were different between medical and surgical departments (results not shown).

**Figure 1:**
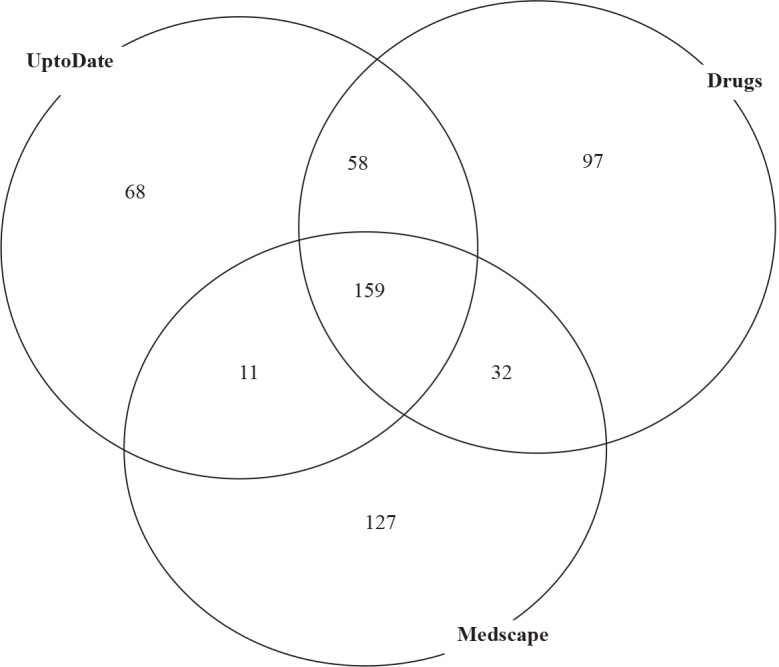
Venn diagram of the distribution of unique interactions as reported by UptoDate, Drugs, and Medscape.

Of the 1335 interactions, 552 were unique, meaning that one particular interaction between antibiotic A and drug D was documented ≥1 and reported by one, two, or all three databases. The distribution of these unique interactions as reported by the databases is shown in Figure 1. In the figure, the total count of interactions is 1335. A unique interaction is defined as one particular interaction between antibiotic A and drug D that was documented at least once, but counted as 1 no matter how many times it appeared.

UptoDate reported 296, Drugs reported 346, and Medscape reported 329 of these unique interactions. One hundred fifty-nine of these unique 552 interactions were reported by all databases.

**Figure 2:**
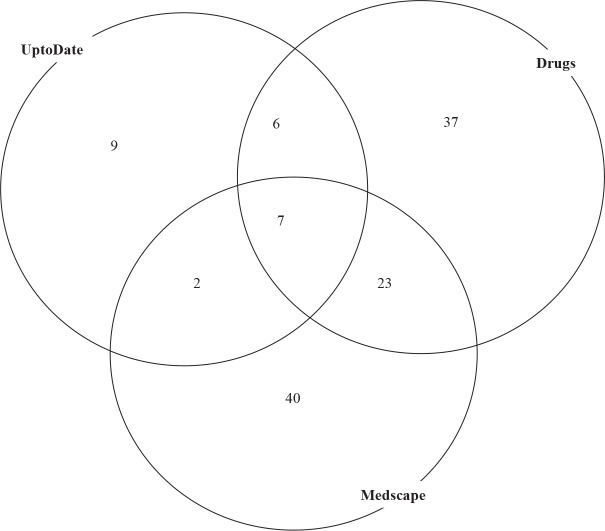
Venn diagram of the highest level of unique interactions as reported by UptoDate, Drugs, and Medscape.

The highest level of interaction is reported with X, major-contraindicated, and contraindicated by UptoDate, Drugs, and Medscape, respectively. The distribution of the highest level of unique interactions as reported by the databases is shown in Figure 2. UptoDate reported 24, Drugs reported 73, and Medscape reported 72 of the highest level of unique interactions. Seven interactions in this category were reported by all databases. Highest level of interaction is reported with X, major-contraindicated, and contraindicated by UptoDate, Drugs, and Medscape, respectively. UptoDate reported 24 (9+7+6+2), Drugs reported 73 (37+23+7+6), and Medscape reported 72 (40+23+7+2) of these highest level of unique interactions. Seven interactions in this category were reported by all databases.

## 4. Discussion

Extensive research and accumulating evidence raised awareness in current medical practice of drug interactions and their possible outcomes. Particularly in those patients with comorbidities treated with multiple drugs, a detailed interrogation of possible drug interactions must be acknowledged as a priority. The consequences of drug interactions of all levels concerns health care professionals in every setting. However, reduction in plasma level due to excessive metabolism or increase to toxic concentrations are only two examples that cannot be tolerated in the treatment of infectious diseases. This study was primarily undertaken to evaluate the main characteristics of antibiotic use and how these affect possible drug interactions related thereto. A specific emphasis was put on comparing surgical and medical departments in order to understand the variation in practice and identify the current problems. A nationwide antibiotic restriction program (NARP) was released in 2003 in Turkey (Official Gazette of the Republic of Turkey, Feb 1, 2003). This compulsory program aimed to reduce hospital antimicrobial use by mandating preauthorization from an infectious disease specialist for the use of some several broad-spectrum antibiotics. However, most of the antibiotic decision-making in hospitals takes place with no direct input from an infectious disease specialist mainly due to the insufficient number of experts in hospitals. Hence, rates of resistance indicate that antimicrobial consumption is still not as strictly controlled as required by NARP at the national level and this holds true for Ankara University Hospitals [17]. 

One of the main findings of this study was the prophylactic use of broad-spectrum antibiotics in surgical clinics as documented by the lack of a definitive indication as opposed to medical clinics where the antibiotics were prescribed as part of a targeted therapy. The propensity in surgical clinics in our study was to prescribe ceftriaxone and metronidazole. A similar recent study reported that initial treatment started with metronidazole and cefuroxime in surgical clinics and mostly stepped up to intravenous broad-spectrum agents [18]. The authors confirmed surgical prophylaxis or surgical site infection as the targets of initial antibiotic use [19,20]. Our study was designed to measure the characteristics of initial prescription of antibiotics and to correlate this information with possible drug interactions. Therefore, a detailed analysis related to antibiotic use such as antibiotic exposure days or duration of treatment was not conducted.

Using a slightly different approach, another study from Turkey reported the frequency and potential drug–drug interactions in five different hospitals. Here, the authors showed that more than 25% of all interactions were associated with antimicrobials. In addition, the number of prescribed antimicrobials, as well as prescribed drugs and hospitalization in the university hospital were independent risk factors for developing drug interactions [21].

Both meropenem and ceftriaxone, two of the most prescribed antibiotics by medical and surgical clinics, respectively, have a unique status in NARP. Meropenem requires preauthorization from an infectious disease specialist. Ceftriaxone treatment may be initiated by any specialist if the treatment is limited to 72 h and extended use beyond the 72 h limit also requires authorization by an infectious disease specialist. In other words, other specialists are comparatively less limited when prescribing a rather broad-spectrum antibiotic, ceftriaxone. This information indicates that antibiotic stewardship interventions targeting surgical clinics need to go beyond prophylaxis and require a stronger input from infectious diseases specialists.

Another important finding of this study is that a PPI, pantoprazole, appeared in 63% of all orders in both medical and surgical clinics without a clear indication. Overuse of PPIs has repeatedly been reported by many studies [22,23]. Despite some controversy, evidence has linked PPI use with serious adverse effects such as increased risk of *Clostridium difficile* ( *C. difficile* ) infections, community-acquired and hospital-acquired pneumonia, and andosteoporotic fractures [24–26]. A recent metaanalysis and systematic review of 14 studies showed that antibiotic exposure and PPI use appeared to be the most important risk factors associated with *C. difficile* infection in children [27]. Apart from these serious outcomes, PPIs carry a considerable potential for drug interactions not only based on their potential to alter gastric pH but also because of their different affinities for some of the drug-metabolizing enzymes in the liver [28,29]. The number of orders that included a PPI was not different between medical and surgical clinics. However, a high prescription rate still indicated that all health care professionals should remain vigilant and continue judicious use of not only antibiotics, but also PPIs in hospitalized patients. 

One of the main questions of this study was whether differences in antibiotic use would affect the possible drug interactions as reported by the web-based interaction tools. The immediate challenge was the observation that these tools were not consistent with the potential interactions that they reported. The clinicians often suffer lack of sufficient time in planning a treatment, which does not allow them to check all available resources for possible drug interactions. We, therefore, felt the need to identify the database that is the most efficient in reporting drug interactions so that this particular database could be preferred in the future to check for possible interactions. Two parameters were utilized: 1. How efficient is a database in reporting the interactions that were also reported by other databases? 2. How efficient is a database in reporting the highest level of interactions that were also reported by other databases?

UptoDate was the most efficient in reporting common interactions (159/296) compared to Medscape (159/329) and Drugs (159/346). The efficiency of UptoDate in reporting the highest level of interactions was also the greatest (7/24) compared to Medscape (7/72) and Drugs (7/73). Thus, UptoDate appeared to be somewhat stronger in reporting possible drug interactions.

This study has some limitations such as the lack of confirmation of the reported drug interactions. This is primarily because of the design, where no follow up was planned. Instead, whether the basic characteristics of the initiation of antibiotic use were different between medical and surgical clinics was the main question of this study. However, any possible interaction of a high rank regardless of the database was immediately communicated with the related clinic during the study. Again, whether or not drug regimen was altered by the clinic based on our recommendation was not tracked. 

We believe that the presence of additional health care professionals, such as a trained clinical pharmacist, at the initiation of therapy and perhaps during the course of hospital stay will provide an additional check-point and help to minimize possible drug interactions and other drug-related issues. 

## Informed consent and ethical approval

The study was approved by the Ethics Committee of the Faculty of Medicine of Ankara University (Date: 28.05.2018, Number: 09-588-18) with a waiver of informed consent due to the retrospective nature of the study.
